# Intraoperative Sphenoid Sinus Volume Measurement as an Alternative Technique to Intraoperative Computer Tomography

**DOI:** 10.3390/diagnostics10060350

**Published:** 2020-05-28

**Authors:** Sergei Karpishchenko, Irina Arustamyan, Olga Stancheva, Kirill Sharko, Dmitry Kaplun, Mikhail I. Bogachev

**Affiliations:** 1 ENT Department with Clinic, Pavlov First Saint Petersburg State Medical University, 197022 St Petersburg, Russia; karpischenkos@mail.ru (S.K.); a-irina26@yandex.ru (I.A.); olga.stancheva@yandex.ru (O.S.); 2 Department of Radiology and Radiation Medicine with X-ray and Radiology Units, Pavlov First Saint Petersburg State Medical University, 197022 St Petersburg, Russia; kirillclone@icloud.com; 3 Department of Automation and Control Processes, Saint Petersburg Electrotechnical University “LETI”, 197376 St Petersburg, Russia; 4 Radio Engineering Systems Department, Saint Petersburg Electrotechnical University “LETI”, 197376 St Petersburg, Russia; rogex@yandex.com; 5 Institute of Fundamental Medicine and Biology, Kazan Federal University, 420008 Kazan, Russia

**Keywords:** sphenoid sinus, methylene blue, endoscopy, computer tomography, nasal cavity, neoplasms, paranasal sinuses

## Abstract

Isolated sphenoid sinus disease (ISSD) is where there is a group of pathologies characterized by inflammation in one or both sphenoid sinuses. Although computer tomography (CT)-based 3D reconstruction remains the gold standard among noninvasive approaches to ISSD diagnostics, no standardized techniques for direct intraoperative measurements of the sphenoid sinus volume in ISSD patients have been documented. We suggest a novel technique for the intraoperative measurement of the sphenoid sinus volume. Our technique is based on filling the sinus with 0.01% methylene blue solution after an endoscopic endonasal sphenoidotomy. The proposed technique was applied to 40 ISSD patients during surgery. Obtained intraoperative measurements were compared to noninvasive measurements from 3D reconstructions based on preoperative CT scans. Our results demonstrated that the obtained measurements did not exhibit significant differences exceeding 0.4 cm^3^, with CT-scan-based measurements in 39 out of 40 cases (*p* < 10^−6^, Wilcoxon sign-rank nonparametric test), thus confirming the accuracy of the proposed technique. Disagreements between direct intraoperative and CT-based measurements in a single case have been attributed to the presence of remaining pathological masses in the sinus, which was further confirmed during the secondary check of the operated sinus. Accordingly, we suggest that the agreement between the CT-based and intraoperative volume measurements can be used as an indicator of the successful elimination of all pathological masses from the sinus without having to perform an adequate exposure of the entire sphenoid sinus to reduce intraoperative bleeding. The proposed technique is accurate and does not require the involvement of specialized intraoperative CT scanners and avoids additional radiation exposure for the patient during an additional postoperation CT scan to confirm the success of the surgery.

## 1. Introduction

Isolated sphenoid sinus disease (ISSD) is a group of pathologies characterized by the inflammation in either one or both sphenoid sinuses, most of them not of neoplastic but, rather, of inflammatory origin [1]. Although this is a relatively rare pathology occurring in about 1–3% of all paranasal pathological cases [2], its diagnostic is often complicated and delayed by nonspecific or vague symptoms. Serious complications such as cranial nerve deficits, visual alteration, and visual loss, as well as pain and numbness, can lead to potentially devastating outcomes if not treated at the early stages. In addition to the sphenoid sinus itself, complications often affect neighboring structures such as cranial nerves II, III, IV, V, and VI; the dura mater; pituitary gland; cavernous sinus; internal carotid artery; sphenopalatine ganglion; sphenopalatine artery; and pterygoid canal [3].

The sphenoid sinus is the deepest air-filled space in the human head and can be directly assessed only during operation. In recent practice, no methods for the direct measurement of the sphenoid sinus volume in ISSD patients have been documented. Nonenhanced multidetector computed tomography (CT) remains the gold standard for the noninvasive analysis of the paranasal sinuses status [4]. Innovations in 3D visual imaging technology, including enhanced technologies for anatomic structures mapping, led to the development of the functional endoscopic sinus surgery (FESS) [5]. For endonasal neurosurgery, it is essential to gain access to accurate 3D mapping due to strongly individual anatomic features and pneumatization properties that should be taken into account to avoid the risk of important neurovascular structures being damaged. Thus, objective measurement techniques are essential for the accurate assessment of the anatomic features of the sphenoid sinus, including pathological cases observed in ISSD patients.

The CT scanning facilitated with sophisticated segmentation and 3D reconstruction algorithms, although not completely automated to date, provides a powerful tool for an early noninvasive diagnostic of sphenoid sinus abnormalities. It is essential for adequate planning of the surgical approach [6,7,8]. In addition to surgery planning, it is also essential to confirm the successful cleaning of the sinus during endoscopic sphenoidotomy [9]. This confirmation is, in turn, often complicated by the anatomical features, such as a deep lateral recess of the sphenoid sinus, especially characteristic of hyperpneumatized sinuses [10]. In such cases, even with the help of angled endoscopes, the surgeon does not always obtain a complete overview of the sinus. Accordingly, visual confirmation of the successful removal of all fungal masses is not always possible, especially when it comes to the fungal balls and/or mycotic lesions located in the lateral recess of the sphenoid sinus [11]. In turn, remaining fungal masses lead to ISSD relapse in about 70% of cases [12]. During malignant tumor operations, the surgeon typically obtains a better overview of the lateral wall of the sphenoid after removing the maxillary sinus posterior wall. However, this kind of intervention is, in most cases, excessive during operations of patients with fungal sphenoiditis and could be avoided if there is another way to validate the complete removal of the fungal masses.

Here, we propose a rather simple alternative approach to the direct intraoperative measurement of the sphenoid sinus volume. We investigated whether the intraoperative sphenoid sinus volume measurement with methylene blue could be an alternative technique to the intraoperative CT sphenoid sinus volume measurement. By comparing the intraoperative volume measurement to the preoperative CT-based volume estimate, the surgeon can confirm the efficacy of the pathological masses’ resection. This way, the course of the operation can be better controlled, without having to perform an adequate exposure of the entire sphenoid sinus to reduce intraoperative bleeding.

## 2. Materials and Methods

We investigated patients diagnosed with ISSD in the ENT Department at Pavlov First Saint Petersburg State Medical University, Russia. The study was approved by the Ethics Committee of the Pavlov First Saint Petersburg State Medical University (17 Nov 2017, protocol No. 11). Written informed consent was obtained from all participants.

The initial diagnosis was based on CT scans obtained between December 2017 and January 2019. Exclusion criteria included any conditions that could affect the development of isolated sphenoid sinusitis, such as diabetes or immunodeficiency, as well as benign tumors and malignant lesions affecting the sphenoid sinus or the sphenoid bone and opacification of the other sinuses.

3D model reconstructions of the sphenoid sinuses were obtained from these preoperative CT scans using the ITK-SNAP open-source software package (University of Pennsylvania, Philadelphia, PA, USA), a popular tool widely used for the semiautomatic segmentation of medical images using active contour methods and for the structural selection of the areas of interest. Volumes of both sinuses were automatically calculated by VAM software version 2.8.3 (Canfield Scientific, Parsippany, NJ, USA), a widely used medical image processing and visualization tool capable of the quantitative characterization of selected 3D structures in medical images [13].

All patients underwent intraoperative sphenoid sinus volume measurements with the use of a methylene blue solution. Measurements were performed on the patients in a supine position, when the natural ostium is located in the upper part of the sinus and, thus, can be filled by medical fluids. Prior to measurements, blood and other fluids were removed from the sphenoid sinuses. Measurements were performed by filling the sinuses with 0.01% methylene blue with the help of an insulin syringe that was inserted directly into the sinuses that did not require any additional needles or nozzles. Filling was done by 1-mL portions until the sinuses were entirely filled. The total volume of the liquid required to fill the sinuses were recorded and further used as a volume estimate. Finally, the injected liquid was removed from the sinuses. Methylene blue is not only used as a contrast agent but, also, as a gel in photodynamic therapy for the hidradenitis suppurativa and as an independent fluid with a local antifungal effect [14,15]. Methylene blue is characterized by an acute toxicity estimate of about 2 mg/kg, and its 0.01% solution is approved for topical application according to Regulation (EC) No. 1907/2006, Annex II.

The agreement between the volume of the injected contrast fluid and the previously obtained volume using the CT scan-based approach indicated that all the pathological masses had been successfully eliminated from the sphenoid sinuses. Statistical comparison between the CT-based and the intraoperative volume measurements was performed using the Wilcoxon signed-rank nonparametric test.

## 3. Results

A total of 40 patients were included. Of them, 33 were women, and seven were men. The average age of the patients was 39 years, mainly Caucasian. Most patients exhibited neurological symptoms such as unclear but specifically localized headaches.

Unilateral endoscopic endonasal sphenoidotomy was performed in 88% of patients, whereas 4% required an opening of the sphenoid sinus through a transseptal approach, and 8% required bilateral endoscopic sphenoidotomy. In all cases, surgical intervention was performed during treatment under endoscopic observation and the control. Of the 40 patients, 6 had polyps, 7 had fungal balls in their sphenoid sinuses, 6 had a combination of fungal sinusitis and purulent processes, 10 exhibited cysts of the sphenoid sinuses, and 11 had an ongoing purulent process.

**Figure 1:**
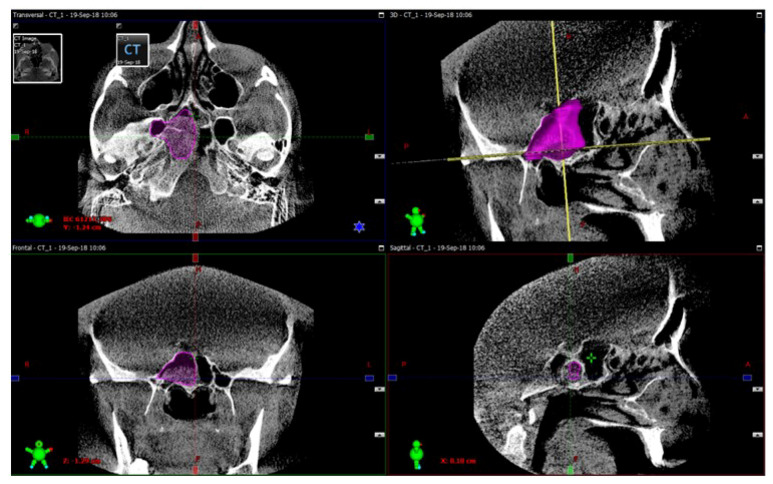
Sphenoid sinus volume measurement procedure based on computed tomography (CT) scanning followed by 3D reconstruction. The opacification of the sphenoid sinus is marked in purple. The exemplified opacified sinus volume highlighted by violet in the figure equals 9.4 cm^3^. The green color denotes the scan area; the red color marks isocenter position; the blue color is horizontal axis; while the yellow line denotes the head anatomical axis.

**Figure 2:**
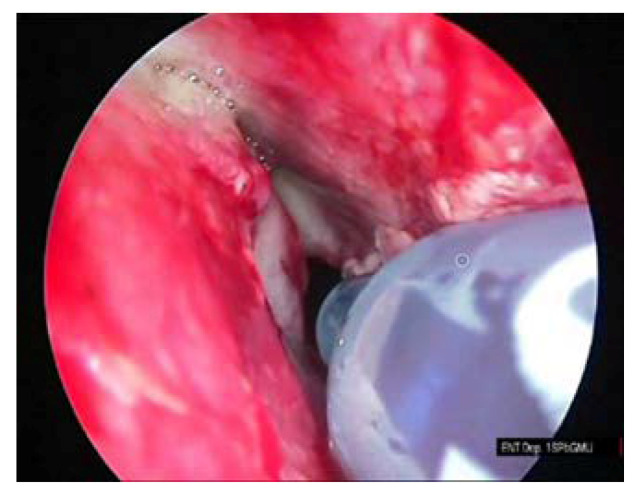
Filling of the sphenoid sinus with 0.01% methylene blue solution with the insulin syringe.

**Figure 3:**
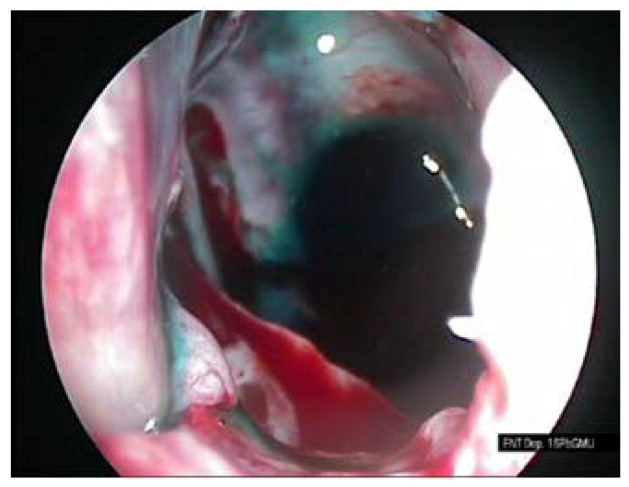
Colored mucosa of the sphenoid sinus.

**Figure 4:**
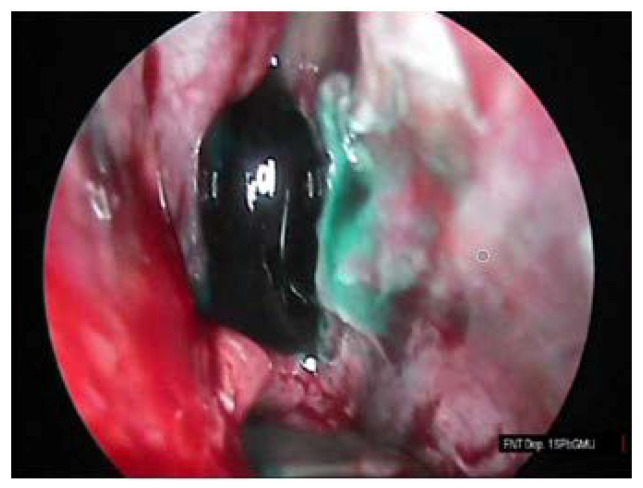
Sphenoid sinus filled with methylene blue solution.

The procedure of the sphenoid sinus volume based on CT scanning followed by 3D reconstruction is illustrated in Figure 1. The corresponding intraoperative measurement procedures are illustrated in Figure 2, Figure 3 and Figure 4.

The results of the intraoperative measurements, as well as the volume estimates obtained from the CT scan-based reconstructions, are summarized in Table 1.

**Figure 5:**
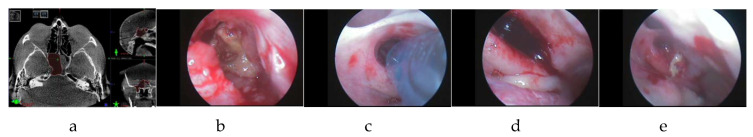
Detailed illustration of a single case where the first measurement indicated that not all fungal masses were removed. After elimination of the fungal masses, a second control measurement was performed. (**a**) Measurement of the sphenoid sinus volume from the CT-scan 3D reconstruction (green color is scan area). (**b**) Fungal ball in the sphenoid sinus. (**c**) Filling in the sinus with the methylene blue solution. (**d**) The sinus filled with the contrast fluid during the initial measurement, indicating a 6.9-cm^3^ volume that is well below the 8.8 cm^3^ expected from the preoperation CT scan. (**e**) After the second surgical revision and several irrigations, remaining fungal masses were eliminated, indicated by the 8.5 cm^3^ volume obtained in the control measurement.

No significant differences in the sphenoid sinus volume were noticed between methylene blue and the preoperative CT scan in 39/40 cases (*p* < 10^−6^, Wilcoxon signed-rank nonparametric test). In one single case (denoted as patient 40 in Table 1), a significant discrepancy between the CT-based and the intraoperative volumes measurements of nearly 2 cm^3^ was observed. This unlikely discrepancy suggested the possible presence of remaining fungal masses in the sinus. The operation was continued to visualize the deep sections of the sphenoid, eventually leading to the confirmation of the above hypothesis. The remaining fungal masses were then successfully eliminated by repetitive irrigation. Another volume measurement confirmed that the pathological masses had been successfully removed, indicated by a small discrepancy of 0.3 cm^3^ (see Figure 5 for this single-case example).

The mean and median of the sphenoid sinus volume were 6.6 cm^3^ and 6.8 cm^3^, respectively, based on CT measurements. We found that smaller-volume discrepancies of less than 0.4 cm^3^ could be typically attributed to the edema of the sinus mucosa and do not indicate the existence of residual pathological masses in the sinus.

## 4. Discussion

The sphenoid sinus is the deepest air-filled space in the human head, connected with the nose cavity through the natural ostium located in its frontal wall. Historically, early noninvasive methods to assess the anatomy and measure the parameters of the sphenoid sinus volume were based on radiography, although they had limited accuracy [16]. Later methods based on two-dimensional CT scans of the sinus volumes were developed. To compensate for the unavailability of complete 3D reconstructions, they relied upon mathematical models to predict the volume based on a limited number of 2D projections and hypothesized the paranasal sinus volume and other anatomical features. This approach nevertheless had inevitable limitations once there were any anomalies in the sphenoid sinus anatomy, as it did not take into account the possible formations of edema of the mucosa, as well as the volume of bone margins, thus limiting the information provided to the surgeon at both the pre- and postoperative stages. Recently, more advanced techniques that rely upon complete 3D reconstructions based on CT scan-based measurements became available, allowing for better surgery planning [17,18,19,20,21]. A recent approach based on the computational fluid dynamics (CFD) model requires double CT scan-based validation and does not provide measurements of one specific sinus but of all pneumatized structures at the same time [22].

In our approach, first, a 3D reconstruction was obtained using a manually controlled CT scan segmentation. Although automated segmentation algorithms provide a first-order approximation, in practical scenarios, they experience inevitable errors due to scan quality, resulting in up to 15% discrepancies in the measured sinus volume compared to the manual segmentation [23]. Thus, to date, automated solutions could be used as support systems providing a first-order approximation only, while requiring manual corrections at later stages. At the correction stage, the operator is required to manually examine the images, meticulously, side-image by side-image, to extract the outlines of the target structures and make proper editing adjustments to eliminate the local inaccuracies. Further improvements of the automated segmentation could be achieved with advanced noise reduction [24,25] or multilevel CT scan processing and adaptive object selection algorithms with decision-making based on improved a posteriori statistics, although these algorithms require considerably more computational resources to obtain these statistics in advance [26,27,28]. To date, manual segmentation provides a higher accuracy, although requiring additional activity and more qualified CT scan operators.

The CT scan-based measurements are used for operation planning. During the operation, after the removal of pathological masses from the sphenoid sinus, the intraoperative measurement is performed and compared against the CT scan-based measurement.

In contrast, significant disagreements between the direct intraoperative and previously obtained CT-based measurements immediately inform the surgeon that not all pathological masses have been removed from the sinus, enabling a necessary correction during an operation. Another condition that can affect the imbalance in the volumes includes the edema of the mucosa of the sphenoid sinus. Based on this knowledge, the surgeon proceeds with the revision of the sphenoid sinus and removes the remaining masses, followed by a control measurement if required.

It is important to note that filling the sphenoid sinus with 0.01% methylene blue solution following the removal of fungal masses during operation has another advantage due to its antifungal activity [29], given that the fungal ball is the most common cause of ISSD development in nonimmunodeficient patients [30]. Once the measurement indicates a complete removal of all fungal pieces, due to the antifungal properties of the methylene blue, there is no need for further washing of the sinus with some other antiseptic solution. Accordingly, in addition to the measurement, the proposed approach also acts as disinfection, thus potentially reducing the risk of recurrent inflammation. The proposed technique is easy to use, and the materials required for measurement are generally available and inexpensive.

Although an allergy to methylene blue has been rarely reported, prior to its topical application, a simple test based on putting a single droplet of the 0.01% methylene blue solution at the threshold of the nasal cavity is performed prior to the application of the method. Methylene blue is widely used in mucociliary clearance testing in all populations, including pregnant women and children [31].

Our results also indicate that the affected sinus volume in the ISSD patients is typically larger than previously reported values in healthy subjects, with the median value of 6.8 cm^3^, compared to previous reports of 3.4 cm^3^ in Asian [32] and 4.0 cm^3^ in Caucasian [33] populations, as well as results of more local studies reporting 5.3 cm^3^ in German [34] and 3.5 cm^3^ in Spanish [35] populations. So far, possible reasons for the development of isolated sphenoiditis have not been fully determined. Additional research of the anomalous sinus volume in ISSD patients may be beneficial for a better understanding of the causes of the underlying disease.

## 5. Conclusions

To summarize, we proposed a novel technique for the intraoperative measurement of the sphenoid sinus volume. Our technique was based on filling the sinus with 0.01% methylene blue solution after the endoscopic endonasal sphenoidotomy. The proposed technique was applied to 40 ISSD patients during surgery. Obtained intraoperative measurements were compared against noninvasive measurements from 3D reconstructions based on CT scans obtained at the preoperative stage.

We demonstrated explicitly that the proposed technique is capable of the direct intraoperative measurement of the sphenoid sinus volume, which is essential for the surgeon to control the efficacy during the operation. The obtained measurements did not exhibit any significant differences from the CT scan-based measurements, thus confirming the accuracy of the proposed technique.

The obtained measurements have been compared against preoperative CT scan-based estimates, confirming the efficacy of the surgery during the operation. Disagreements between direct intraoperative and CT-based measurements immediately inform the surgeon that not all pathological masses have been removed from the sinus, enabling a necessary correction in the course of the operation. The proposed technique does not require the involvement of specialized intraoperative CT scanners and allows one to avoid a redundant radiation dose for the patient during an additional postoperation CT scan used to confirm the success of the surgery.

Altogether, complementary information obtained from two sources, including the 3D preoperative CT-based models and the proposed intraoperative measurement technique, could facilitate the improved diagnostics of ISSD-associated pathologies and enhance the surgeon’s knowledge during operation.

## Figures and Tables

**Table 1:** Results of the intraoperative sphenoid sinus volume measurements and their computed tomography (CT) scan-based estimates in 40 patients.

Patient No.	CT-based Volume Estimate (cm^3^)	Intraoperative Volume Measurement (cm^3^)	Discrepancy (cm^3^)
1	1.8	1.6	0.2
2	2.7	2.4	0.3
3	3.4	3.3	0.1
4	3.7	3.7	0
5	4.3	4.2	0.1
6	6.4	6.2	0.2
7	7	6.9	0.1
8	7.3	7	0.3
9	7.7	7.5	0.2
10	8	7.8	0.2
11	8.4	8.4	0
12	8.5	8.2	0.3
13	8.6	8.4	0.2
14	8.8	8.4	0.4
15	10	9.9	0.1
16	10.1	9.8	0.2
17	11.4	11	0.4
18	6	5.9	0.1
19	12.5	12.3	0.2
20	7.4	7.4	0
21	2.5	2.2	0.3
22	9.6	9.4	0.2
23	6.7	6.5	0.2
24	6.4	6.2	0.2
25	1.4	1.4	0
26	3.5	3.3	0.2
27	3.8	3.8	0
28	5.4	5.2	0.2
29	10.1	10	0.1
30	3.5	3.2	0.3
31	9.5	9.4	0.1
32	6.8	6.5	0.3
33	10.4	10.1	0.3
34	5.5	5.5	0
35	1.2	1	0.2
36	6.9	6.7	0.2
37	10.2	10	0.2
38	2.6	2.5	0.1
39	6.5	6.4	0.1
40 *	8.8	6.9	1.9
40 **	8.8	8.5	0.3

* First measurement, and ** second measurement after removal of the remaining fungal masses.
